# Reinforcement of Polylactic Acid Parts Manufactured Additively by Material Extrusion Method by Adding Monofilament Polyester Mesh Interlayers

**DOI:** 10.3390/polym17091191

**Published:** 2025-04-27

**Authors:** Mumin Tutar

**Affiliations:** Machine and Metal Technologies Department, Vocational School of Yenisehir Ibrahim Orhan, Bursa Uludag University, 16900 Bursa, Türkiye; mumintutar@uludag.edu.tr

**Keywords:** additive manufacturing, fused filament fabrication, reinforcement, polyester monofilament mesh

## Abstract

This study investigates the use of monofilament polyester mesh interlayers to enhance the mechanical performance of PLA parts produced by fused filament fabrication (FFF). Through tensile and bending tests conducted on samples manufactured with varying numbers of reinforcement layers (0, 1, 2) and extrusion temperatures (210 °C, 230 °C, 250 °C), it was determined that extrusion temperature significantly influences mechanical properties; low temperatures led to insufficient adhesion issues, while increasing temperatures generally improved strength. It was also found that polyester mesh reinforcement particularly increased tensile strength at low temperatures, but this effect diminished or became negative at higher temperatures. In conclusion, this research suggests that the incorporation of reinforcement mesh interlayers in FFF offers a promising hybrid approach to improve mechanical properties with proper parameter selection.

## 1. Introduction

Additive manufacturing via a material extrusion process, which is well known as fused filament fabrication (FFF) or fused deposition modeling (FDM), is enjoying a widespread growth in popularity in current years due to its versatility, capacity for manufacturing complicated geometries, and cost savings. In this process, plastic melted in a heated nozzle is pushed on a heated platform and is built in a sequential pattern of layers, producing complicated geometries which cannot be made using traditional manufacturing [1,2,3,4,5,6].

Apart from these advantages of the material extrusion-based additive manufacturing process, there exist a few drawbacks as well. One such industry issue is the mechanical properties of the parts, which can reduce due to a number of these flaws, such as interlayer adhesion incompletion and anisotropic material properties. Such flaws result in lower durability and strength, which hinder utilization of additively developed parts in critical applications [7,8].

The incorporation of reinforcement fibers such as carbon, glass, etc., in the FFF process allows the production of high-performance composite materials. Studies have shown that such composites possess better mechanical properties compared to their unreinforced equivalents. For example, studies have shown that carbon fiber-reinforced polymers produced using FFF have better tensile and flexural strength and therefore are apt for applications requiring high structural integrity [9,10,11,12]. Secondly, the ability to control the orientation of the fibers during production allows them to design the mechanical performance of the part according to the specific load conditions to which the final part will be subjected [12,13,14].

One of the main advantages of using FFF to produce fiber-reinforced composites is the design freedom it offers. Compared to traditional manufacturing with tooling or molds, FFF offers the potential for the production of complex geometries and internal structures. This is particularly precious in scenarios in which reducing weight is of paramount interest, such as in aircraft component manufacturing, in which every gram of weight reduction translates directly into a major fuel cost reduction. Secondly, FFF technology, being a technology based on a process of adding material in a sequential, layer-by-layer process, allows for integration of reinforcing material directly in the part being printed, which can lead to more homogeneous material loading and improved stress-bearing capacity [15,16,17,18].

The fiber or mesh material used in manufacturing composite materials, which have usage in the machinery and construction industries, consists of hand lay-up spread between plies. In such a case, inclusion of reinforcing material in the shape of, say, monofilament polyester mesh, can be a fruitful solution for improving the mechanical properties of such polymeric materials. Through interlayer inclusions of mesh in developed structure, interlayer adhesion and structural rigidity can be improved, which can overcome drawbacks of traditional polymeric additive manufacturing [4,19,20].

The use of polyester mesh interlayers is not only for inducing mechanical part printing but is a part of broader interest in manufacturing for sustainability. The inclusion of recycled materials and bio-based materials in additive manufacturing is being viewed as another path for reducing environmental footprints and aiding the concepts of a globalized economy. Polyester is a widely consumed synthetic polymeric material which can be recycled and reutilized and undergoes a more sustainable manufacturing process. Secondly, using mesh interlayers can potentially make manufacturing zero-strength lost-weight structures a possibility. This is of great interest in industries such as aeronautics and automobiles, in which reducing weight is of great interest for efficiency and functionality [20,21].

Aside from mechanical reinforcing, polyester mesh interlayers may contribute toward impacting the rheological and temperature material properties of printed parts. The mesh may alter the release of heat in the process of being extruded, which may lead to improved thermal stability and warpage prevention of the printed part. Interdependencies between material properties, parameters of extrusion, and introduction of interlayers of mesh should be identified in the quest of trying to make an attempt to improve the process of additive manufacturing, as well as the desired end-product properties [4,5].

The exploration of combined manufacturing technologies in a hybrid of an additive manufacturing process and a conventional manufacturing process is another avenue this study attempts. With a blend of technologies, there is a chance of creating greater functionality in a resulting composite. The integration of polyester mesh interlayers in material extrusion is an ideal example of a combined process in so far as it combines the freedom of an additive manufacturing process and the structural advantage of a conventional reinforcing process [8,21,22].

This study investigated the impact of monofilament polyester mesh interlayers on the mechanical performance of polymer components that are produced using material extrusion. The experimental design methods were aimed at explaining the impact of the mesh interlayers on the mechanical performance of the produced part and the functionality of the printed components. The findings are expected to contribute to the existing knowledge in additive manufacturing process optimization and the production of high-performance polymer components for different applications.

In short, the improvement of additively manufactured polymer parts using monofilament polyester mesh interlayers is a main hybrid approach to additive manufacturing. Not only does the new process avoid the limitations of traditional polymer additive manufacturing, but it also follows the overall trends of hybrid manufacturing and sustainability. Throughout the course of this study, it is conceivable that such innovation will achieve the potential of faster, stronger, and more environmentally friendly manufacturing processes and perhaps broaden the applications of additive manufacturing across various industries.

## 2. Materials and Methods

Polylactic acid (PLA) is a biodegradable thermoplastic polymer that has gained significant attention for its environmental advantages. Its sustainability and biodegradability make it an attractive material for a wide range of engineering and structural applications. Although PLA generally exhibits a lower molecular weight compared to many conventional polymers and composites, its cost-effectiveness and renewability contribute to its growing use in various industries [11]. In this study, the extrusion filament material is high-quality and precision Ultrafuse PLA (Ludwigshafen, Germany), which is produced by BASF. Plain weaved polyester monofilament mesh structure with 25 mesh density was used between the selected layers in the production of hybrid fiber-reinforced composites with additive manufacturing to increase strength. Polyester monofilament mesh structures are generally used in the production of food filters and are high-strength and transverse isotropic materials. Sample productions were carried out using a modified direct drive Creality Ender 3Pro (Shenzhen, China), which was also used in previously published studies [12,23,24]. The printing parameters that were kept constant are given in Table 1. The infill density was taken as 100% for all samples. A 0.6 mm nozzle was preferred to fill the gaps between the polyester monofilament mesh more effectively. Since PLA is generally a problem-free material in terms of adhesion to the table, no adhesive or other materials were used for this purpose. After production, the samples were stored in airtight containers containing silica gel to prevent moisture until the test time.

ASTM D638 and D790 standards were followed for tensile and three-point bending tests, respectively. All tests were performed at room temperature with an MNR-100 electronic universal testing machine (Bursa, Türkiye). Table 2 shows the sample dimensions and test parameters given in the standards used.

**Figure 1:**
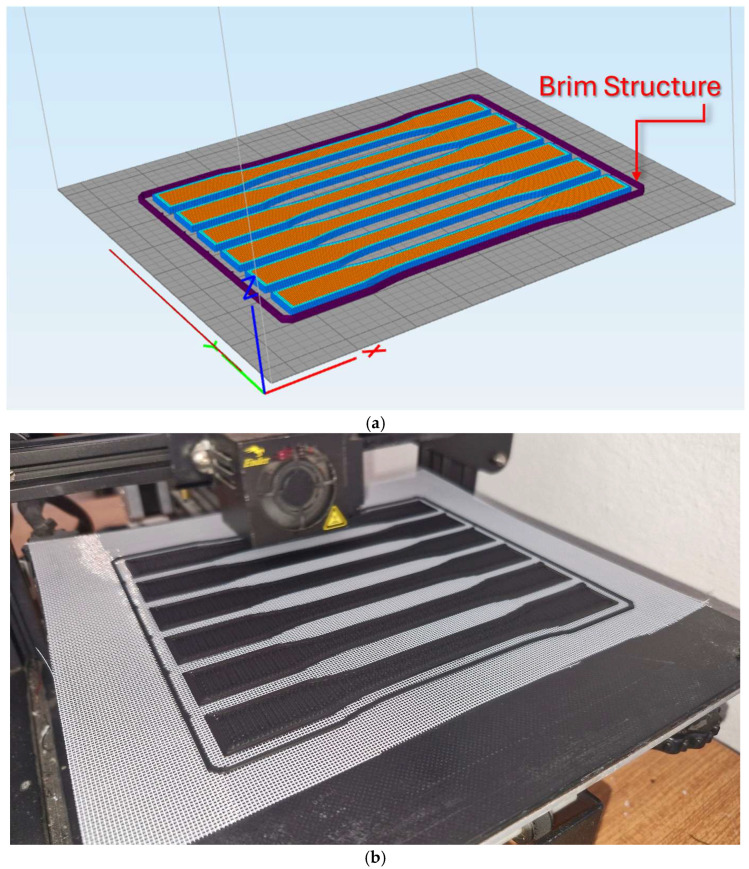
(**a**) Slicer output showing the brim structure. (**b**) Tensile test samples during manufacturing.

By editing the G-codes taken from the Simplify3D V4.1.2 slicing software, it was ensured that the nozzle would rise to a certain height on the Z-axis in the beginning of the layers where the polyester reinforcement would be added and would wait for the production process to continue with a manual command. During this waiting period, polyester mesh structures were manually laid on the surface and production was continued. For the reinforcement polyester not to slip, brim structures were used in 2 rows at a distance of 4 mm from the samples. After the mesh structure was added, the brim structure was first printed, and the slippage of this mesh structure was prevented during the nozzle movement on the sample. This brim structure and the output of the slicing program are shown in Figure 1a. The actual image of the slicer program output while it is being produced on the printer table is shown in Figure 1b.

In order to ensure the same mesh grid for all six samples, firstly, the mesh structures were cut with a proper alignment using a cutting jig. During the addition, it was aligned to the 3D printer’s table by supporting it from the back edge and held with a clip, ensuring that this alignment was always constant. The distances between the samples were calculated by taking into account the number of meshes, and in this way, it was guaranteed that the mesh structures would come to all six samples in the same way. The mesh number 25 refers to the number of yarns in 1 inch. The repeating grid interval is 25.4/25 = 1.016 mm, 5 grid; i.e., 1.016 × 5 = 5.08 mm were left between the samples.

In polymers, viscosity typically determines fluidity and this value decreases with increasing temperature. This behavior is primarily due to enhanced polymer chain mobility and the weakening of intermolecular interactions at higher temperatures. However, factors such as specific chemical structure, side-chain interactions, and the presence of additives can modify this general trend, sometimes leading to non-linear or more complex responses. Therefore, after the reinforcement mesh was added, the printing speed was reduced by half on the first layer and the cooling fan was stopped so that the melted filament material could flow through the gaps of mesh structure and reach the bottom layer and ensure adhesion. In this way, the aim was to reduce the viscosity of the melted filament and provide better adhesion between the layers and the polyester mesh. Another reason for this application is that the added mesh layer is relatively cold, causing the material flowing from the nozzle to cool and solidify rapidly.

The ratio of the volume filled by the mesh polyester structure and the volume of the filament flowing in that layer was calculated using Equation (1). In this calculation, the voids in the layer produced with 100% infill density were not considered. It was calculated that approximately 15.46% of the reinforcement layer was filled by the polyester mesh and the extrusion multiplier in the relevant layer was reduced to 85% to prevent excessive filling as much as the volume filled by the laid polyester mesh structure.

(1) $$\%V=\frac{2n_{mesh}\pi {d_{f}}^{2}L\;}{4{h_{layer}L}^{2}}$$

where *%V* is the ratio of the polyester mesh volume in a layer and the filling volume of the relevant layer, *n_mesh_* is the number of yarns in the *L* width in an axis of the woven mesh structure (25 yarns/inch), *d_f_* is the diameter of a filament (0.2 mm), *L* is the sample length (25.4 mm), and *h_layer_* is the layer height (0.4 mm).

Since the thickness of both tensile and bending samples was 3.2 mm and the constant layer height was 0.4 mm, reinforcement meshes were laid after the fourth layer in single-layer reinforced samples and after the second and sixth layers in two-layer reinforced samples.

In this study, the number of reinforcements and extrusion temperature were selected as experimental parameters to investigate the effect of reinforcement mesh layers on mechanical properties. The experimental design planned as a full factorial is given in Table 3. In order to facilitate the passage of the molten filament flowing from the nozzle to the substrate through the openings of the mesh structure, the extrusion temperature increased to a value (250 °C) above the standard PLA printing temperatures.

## 3. Results and Discussion

### 3.1. Polyester Mesh Layer Reinforcement

**Figure 2:**
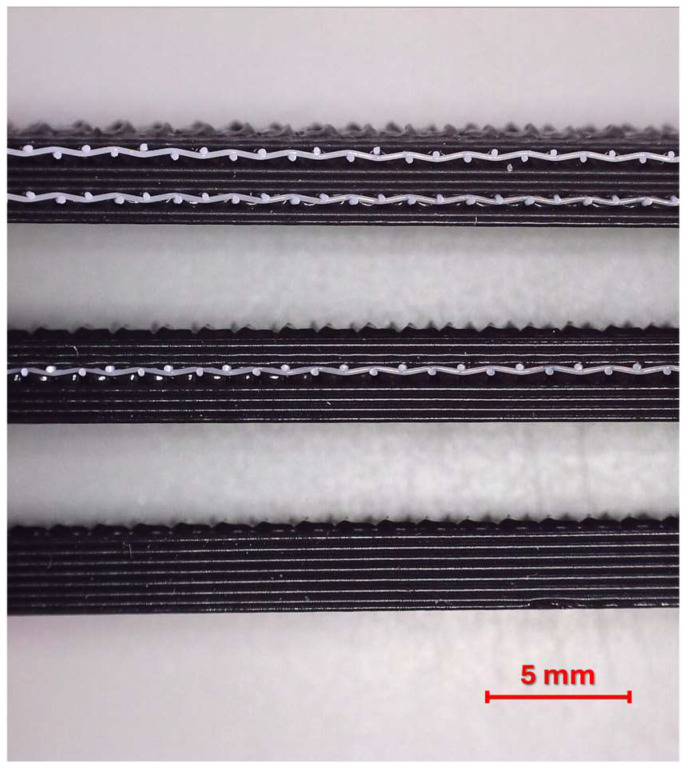
Cross-sectional images of printed samples with different numbers of reinforcement layers.

According to the experimental design listed in Table 3, unreinforced and one- and two-layer reinforced tensile and bending samples were successfully produced. Cross-sectional views of unreinforced and one- and two-layer reinforced samples are shown in Figure 2.

### 3.2. Polyester Reinforcement Tensile Tests

**Figure 3:**
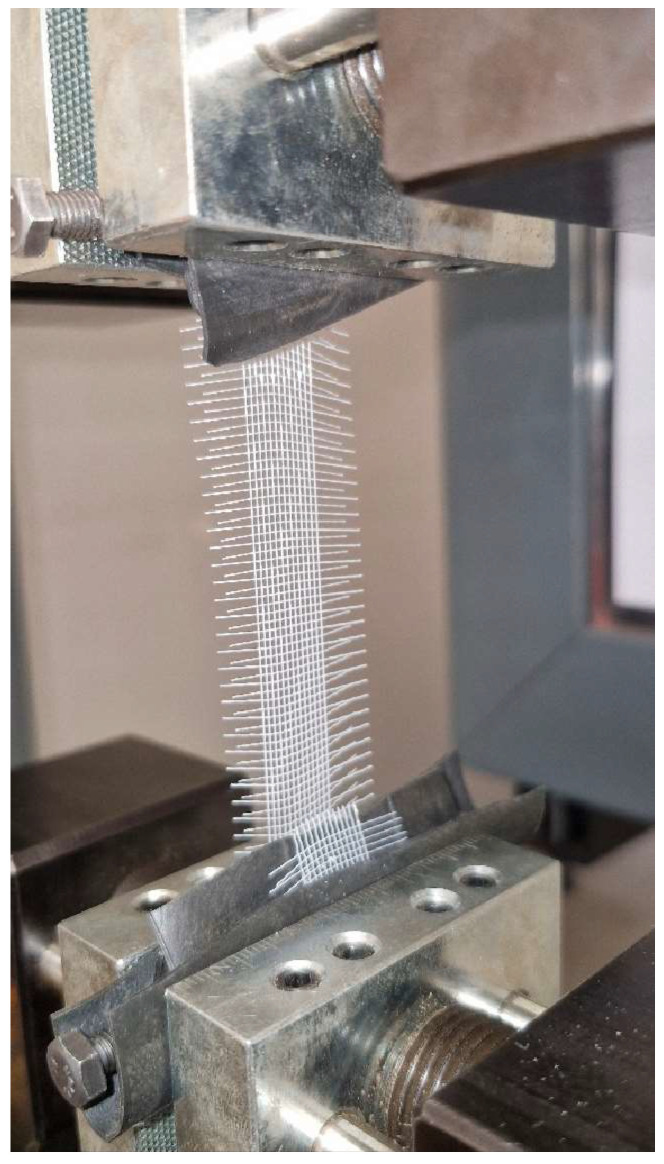
Tensile test of polyester mesh reinforcement structure.

**Figure 4:**
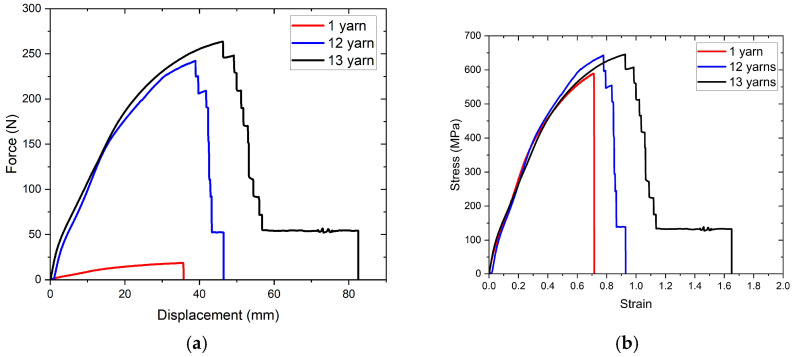
(**a**) Force/displacement and (**b**) engineering stress/strain curves of polyester mesh layers having different numbers of warp yarn.

Tensile tests were performed to determine the mechanical properties of the polyester mesh structure used as a reinforcement element; an image from the tensile tests is given in Figure 3, the force/displacement and engineering stress/strain curves obtained from the tests are given in Figure 4a,b. Mechanical properties calculated using tensile test results are given in Table 4. In carrying out these tests, an extensometer could not be used as it was only suitable for standard tensile test samples and the elongation values were obtained from the crosshead movement. For this reason, while the elongation values could be evaluated comparatively, it was not possible to use them in the tensile modulus calculation.

To understand the effect of the mesh structure produced via plain weaving on the tensile properties, both single yarn and samples containing different numbers of yarns were tested. Thirteen yarns represent the situation corresponding to the width of the tensile and bending samples that will be mentioned in the following sections, and the value of twelve yarns was chosen to observe whether generalizations can be made by collecting the data obtained from separate tests. In other words, the question of whether the load carried by 1 yarn and the load carried by 12 yarns can be reached by adding the load carried by 1 yarn is answered. The tensile strength values calculated using the yarn diameter are close to each other and are compatible with the literature [27,28]. Due to the interaction of the weft and warp yarns in the woven structure, the tensile strength was also affected in the direction of increase with the increase in the number of yarns in the sample. The sudden decreases in the curve observed in the experiments conducted with 12 and 13 yarns, which were not seen in 1 yarn sample, are an indication of the warp yarns breaking. The increase in the breaking elongation with the increase in the number of warp yarns is related to the interlocking of the warp and weft in the woven structure.

In this study, tensile tests of polyester mesh structures were performed on samples that had not undergone any heat treatment. However, Guo et al. exposed monofilaments that were fixed on both sides and left free to heat at 180 °C for 10 min and determined that the mechanical properties of polyester monofilaments deteriorated. Therefore, it should be taken into account that the mechanical properties of the material used as a reinforcement element will also be affected by the heat effect in this study where additive production is carried out [27].

### 3.3. Tensile Tests

**Figure 5:**
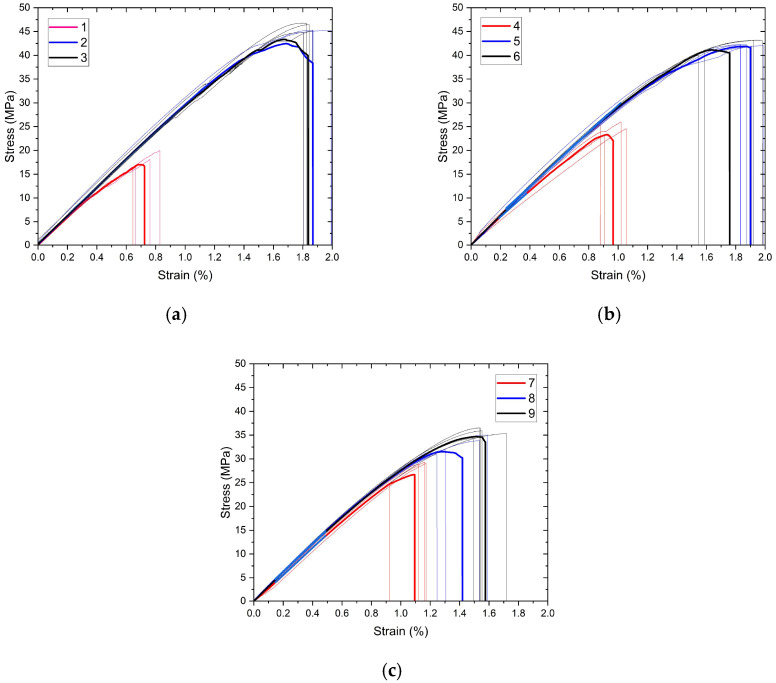
(**a**–**c**) Tensile test curves of samples grouped for extrusion temperature.

**Figure 6:**
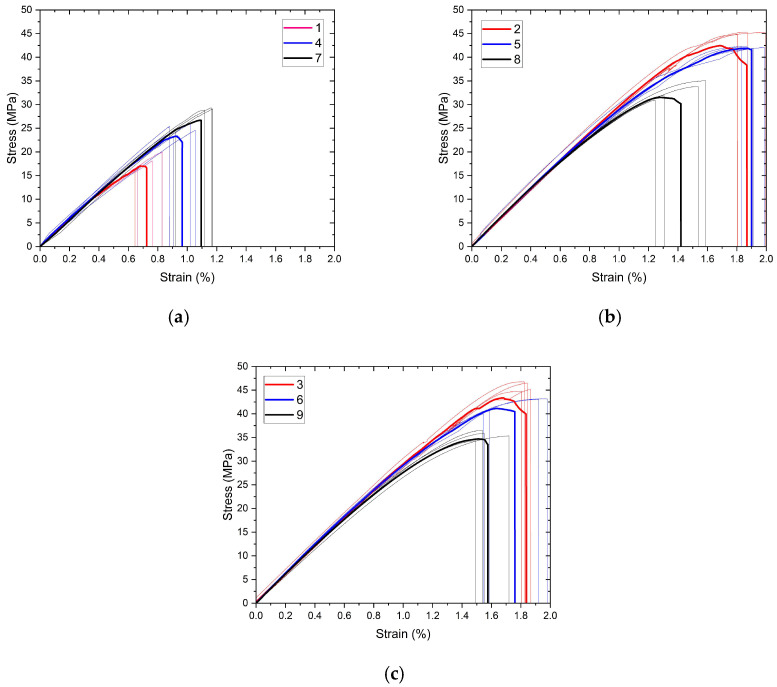
(**a**–**c**) Tensile test curves of samples grouped for the number of reinforcement layers.

The engineering stress/strain curves are grouped in Figure 5 and Figure 6 to evaluate the effects of extrusion temperature and number of reinforcements, respectively. To show the consistency of the findings, repetitive experiment curves and their mathematical average curves (bold curves) are also given together. The tensile properties calculated using these curves are given in Table 5 according to the nomenclature in the experimental design, along with standard deviation values.

**Figure 7:**
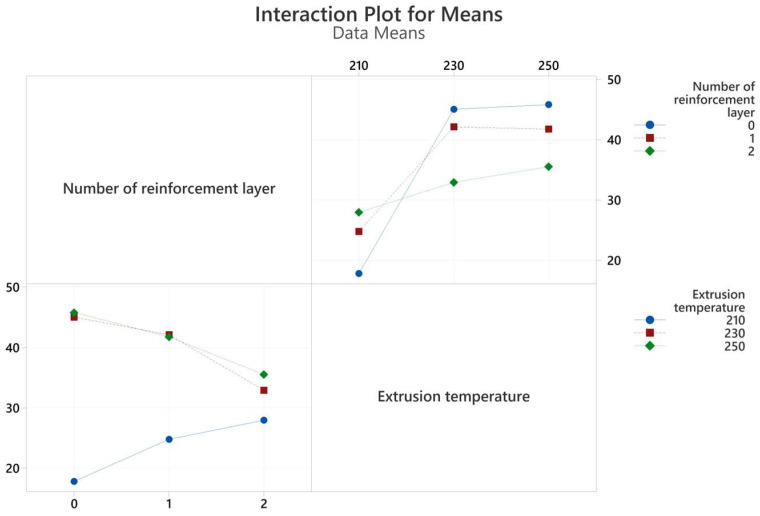
Interaction plots for tensile strength values obtained from tensile tests.

**Figure 8:**
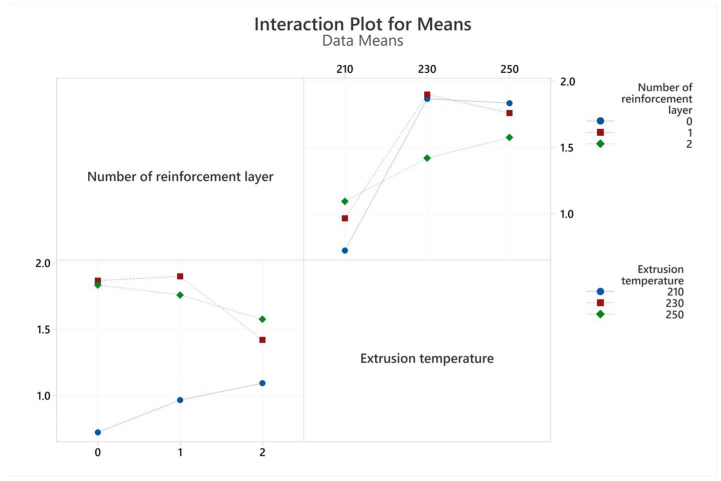
Interaction plots for elongation at break values obtained from tensile tests.

The graph of the interactions of the production parameters with the target functions, tensile strength, and elongation at break values are given in Figure 7 and Figure 8, respectively. An analysis of variance (ANOVA) table was prepared to evaluate the effect of production parameters on the target functions, tensile strength, and elongation at break values. These tables and the calculated contribution percentage values are given in Table 6 and Table 7 together with tensile strength and elongation at break values.

As a result of regression analysis, the R^2^ value obtained for tensile strength was calculated as 79.93 and for elongation at break as 86.82. Although the R^2^ values are statistically quite significant, they were slightly lower for tensile strength due to the interacting and counter-working mechanisms of extrusion temperature and the number of reinforcement layers. For example, while the increase in extrusion temperature decreases the viscosity of the filament material, facilitating flow through the openings of the reinforcement mesh structure and making the structure more robust, it also causes chemical degradation of the PLA material. In addition, an approximately 97% correlation was found between tensile strength and tensile strain. Achieving the targeted composite structure appropriately resulted in increased deformation with increased tensile strength.

The extrusion temperature was the parameter that most affected both tensile strength and elongation at break. The contribution percentage values were calculated as 75.58 and 83.63 for tensile strength and elongation at break, respectively. At the lowest extrusion temperature (210 °C), both strength and elongation values remained very limited for all three reinforcement conditions. This situation can be explained by the insufficient interlayer adhesion of the filament at low temperatures [29]. Due to the rectilinear 90° printing pattern, the adhesion properties in the tensile direction were also quite low. At this temperature value, both tensile strength and elongation at break showed a slight increase as the number of polyester mesh reinforcements increased. With increasing temperature (230 °C), adhesion improved, and consequently, strength and elongation values increased significantly. This increase showed its highest value in the unreinforced sample group, and tensile strength increased approximately three times. However, at this level, the effect of reinforcement was limited due to the increased adhesion capability, so much so that the sample with two reinforcement layers, which showed the highest strength at the lowest temperature value, showed the lowest strength value at the second level of temperature values. A further increase in temperature (250 °C) caused an insignificant increase in tensile strength while causing a decrease in elongation at break. Although the PLA filament material used was a modified material and the printing temperatures were higher than those of basic PLA, the chemical degradation of the PLA material at this temperature value may have caused these results.

When the ANOVA tables in Table 6 and Table 7 are examined, it is seen that the effect of the number of reinforcement layers on the tensile test results remained minimal within the total variation. The reinforcement layers consist of 13 warp yarns in tensile and bending samples. The tensile strength of a woven polyester structure of this width was obtained as approximately 650 MPa. Despite these superior mechanical properties, increasing the number of reinforcement layers showed improvement in mechanical properties only at the lowest temperature level (210 °C). This increase amounted to approximately 60%. At this low temperature level, where adhesion was poor, the primary mechanism affecting the mechanical properties was through the reinforcement layers. However, at increasing temperature values where adhesion improved, increasing the number of reinforcements led to a slight deterioration in mechanical properties. Goh et al. reported that increasing fiber reinforcement negatively affects the interfacial adhesion and reduces the tensile strength [30].

The interface is typically regarded as an intermediate region formed through the interaction between the matrix and the fibers, with a thickness ranging from a single atom to several microns. An effective interfacial layer facilitates strong bonding and efficient stress transfer between the fiber and matrix without causing structural discontinuities, thereby enhancing the composite’s overall performance. Consequently, a thorough investigation of interfacial characteristics is essential. The primary mechanisms governing fiber/matrix interfacial bonding include interdiffusion, electrostatic adhesion, chemical bonding, and mechanical interlocking. In most cases, interfacial adhesion results from a combination of these mechanisms, although one often predominates depending on the specific material system. In this study, strength increase is aimed at forming a mechanical interlocking. However, the adhesion problem between PLA and polyester caused the strength increase to be limited [30,31,32,33].

**Figure 9:**
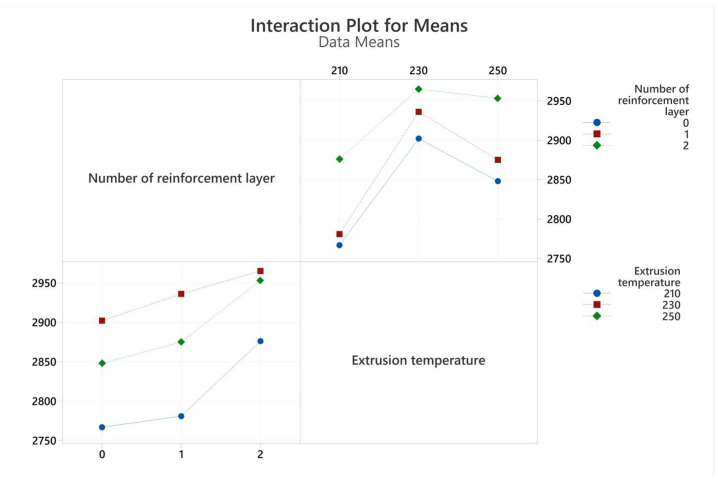
Interaction plots for tensile modulus values obtained from tensile tests.

Tensile modulus values were calculated using mathematical average curves and are shown in Table 5. The interaction plot for tensile modulus values is visualized in Figure 9. The highest tensile modulus value was obtained in the two-layer reinforced sample produced at a 230 °C extrusion temperature. It should be noted that the tensile modulus increased with the increase in the number of reinforcement layers at all extrusion temperature levels. This is clearly related to the higher tensile modulus of the polyester mesh structure compared to PLA. With increasing extrusion temperature, the tensile modulus first increased and then decreased. This situation can be associated with the increase in the heat exposure of the polyester and the properties of the PLA filament going beyond the optimum values with the increasing extrusion temperature [27,30].

### 3.4. Three-Point Bending Tests

**Figure 10:**
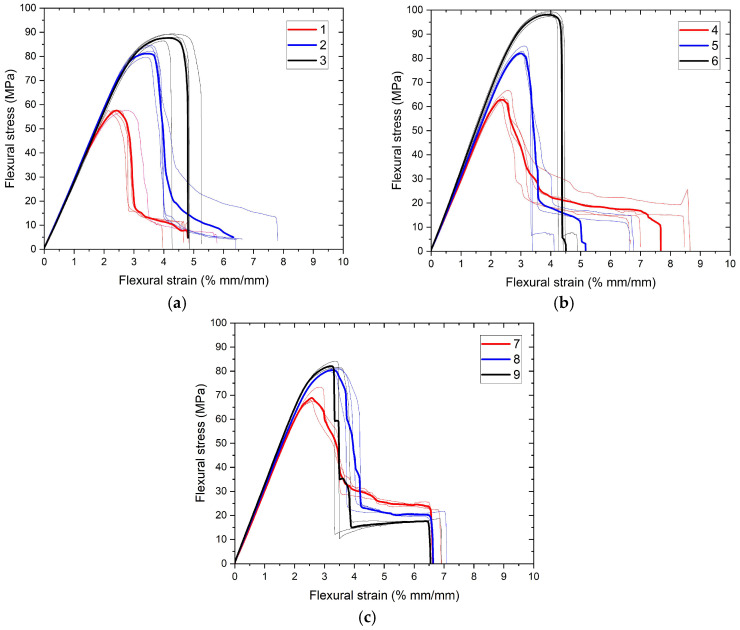
(**a**–**c**) Three-point bending test curves of samples grouped for extrusion temperature.

**Figure 11:**
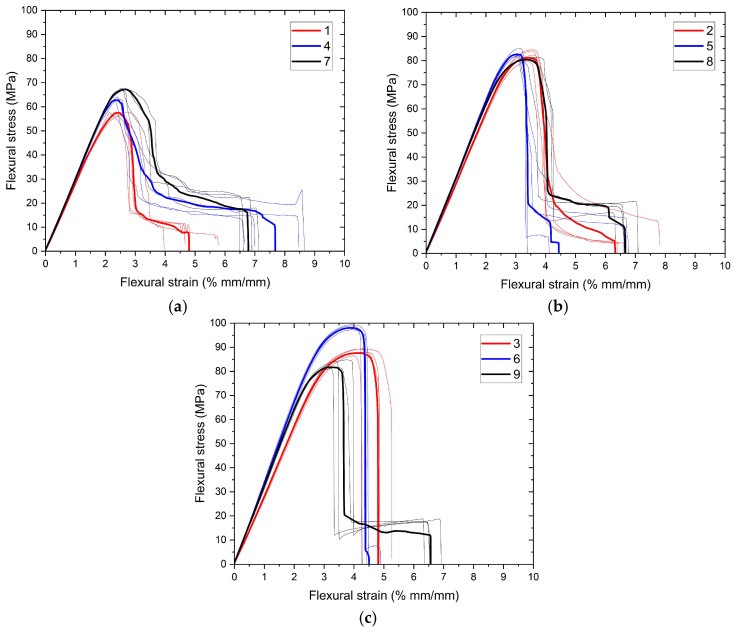
(**a**–**c**) Three-point bending test curves of samples grouped for number of reinforcement layer.

Engineering curves obtained from three-point bending tests are visualized in Figure 10 and Figure 11. As in tensile tests, figures were grouped to evaluate the effects of extrusion temperature and number of reinforcements for bending tests. To visualize the repeatability of the experiments, the averages of the experiments are also plotted (bold curves) in the relevant graphs. As can be seen, the repeatability of the experiments is quite high. This indicates that the production processes are not significantly affected by noise parameters. Flexural strength and flexural strain at the onset of failure were calculated with the obtained data and presented in Table 8 along with standard deviation values.

**Figure 12:**
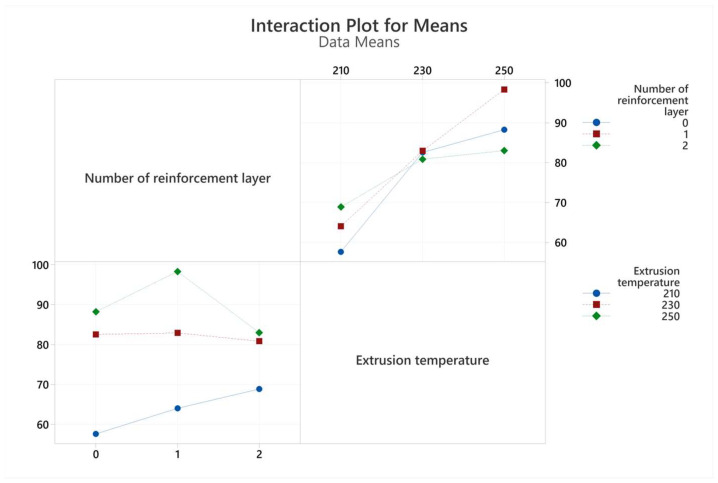
Interaction-plot flexural strength values obtained from three-point bending tests.

**Figure 13:**
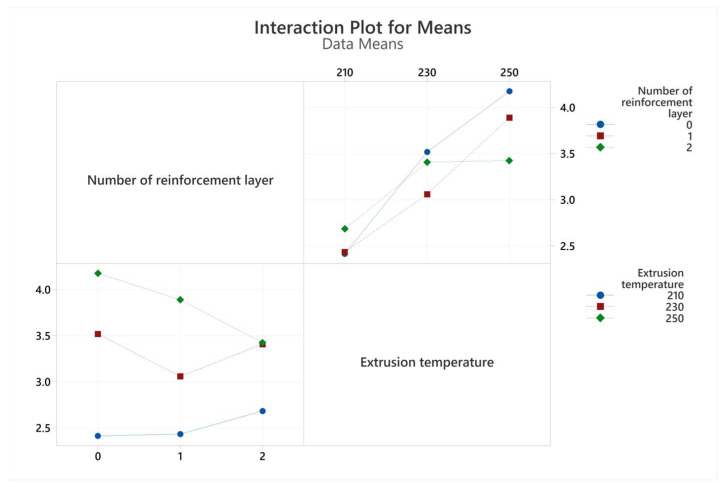
Interaction plots for flexural strain values obtained from three-point bending tests.

The interactions of bending test results with extrusion temperature and number of reinforcements are given in Figure 12 and Figure 13. Analysis of variance for both flexural strength and flexural strain is shown in Table 9 and Table 10. The R^2^ values obtained from regression analyses were calculated as 89.41 and 88.81 for flexural strength and flexural strain, respectively. The best sample for flexural stress was obtained in a single reinforcement layer at an extrusion temperature of 250 °C.

It was determined that extrusion temperature affected flexural strength and flexural strain by 85.43% and 85.62%, respectively (Table 9 and Table 10). Both values increased with increasing temperature. The increase in flexural strength with increasing extrusion temperature occurred at the highest level in the one-layer reinforcement conditions, at approximately 60% (fourth and sixth samples). Increasing the number of reinforcements increased flexural stress at the lowest extrusion temperature level, but this effect was not observed at higher temperatures. This situation can be explained by the improvement of the strengthening mechanism through good interlayer adhesion with the increase in extrusion temperature. The adhesion between polyester fibers and PLA matrix material remained at an insufficient level at all temperature levels. Flexural strain values, like flexural stress, continuously increase with extrusion temperature. However, except for the lowest extrusion temperature, the increasing number of reinforcement layers decreased the flexural strain at break. This situation is also associated with matrix/fiber bonding as indicated in some other studies [34,35,36].

### 3.5. Optical Inspection for Fracture Surfaces

**Figure 14:**
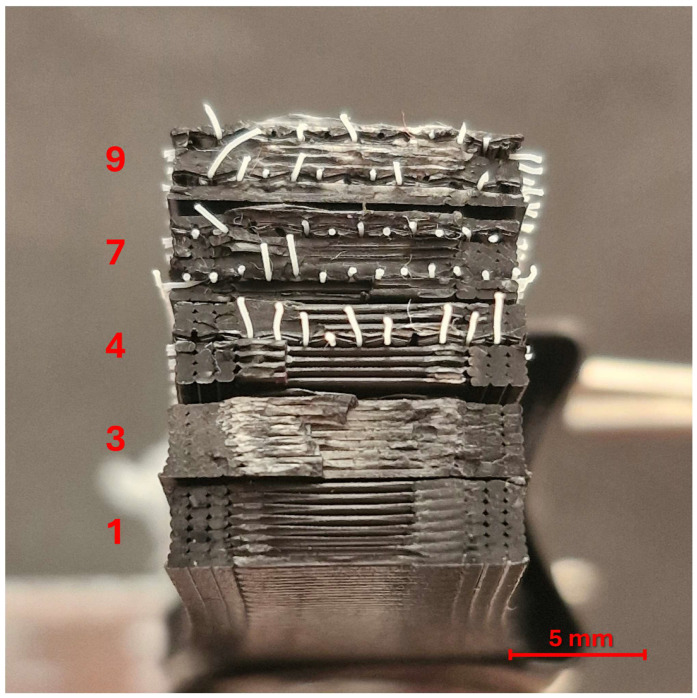
Macro images for fracture surfaces of selected tensile test samples.

**Figure 15:**
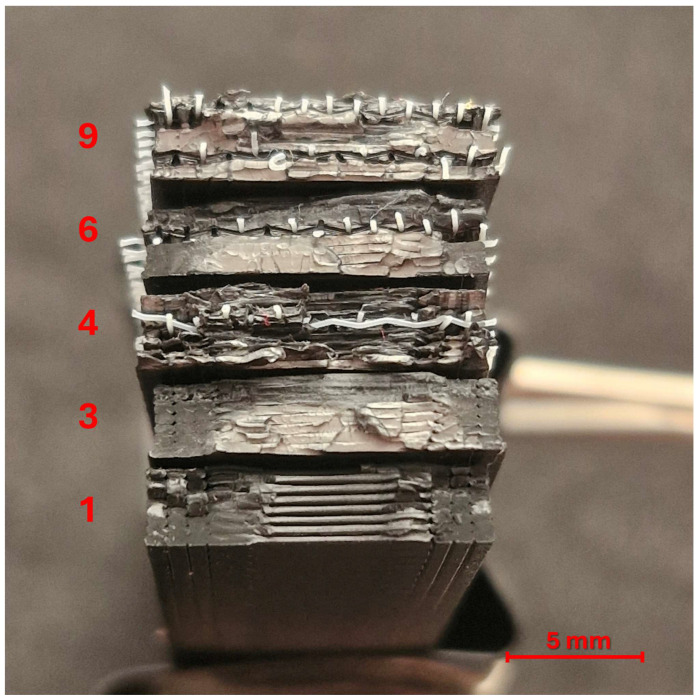
Macro images for fracture surfaces of selected three-point bending test samples.

The macro images of the fracture surfaces of some selected tensile samples in Figure 14 and bending samples in Figure 15 are given along with their experiment numbers. In these images, porous surfaces indicate ductile fracture behaviors, while shiny, smooth surfaces indicate sudden and brittle fractures [37,38,39].

When the fracture surface of sample number 1 (210 °C extrusion temperature, unreinforced), which has the lowest tensile strength among the tensile test samples, is examined, it is seen that a small part parallel to the tensile axis and outside the region forming the three shell outlines shows good adhesion and creates a bearing load. In the middle part of the sample, where the printing pattern is rectilinear 90°, it is seen that the adhesion is not at the desired level in the region perpendicular to the tensile axis, especially in the regions where the printer nozzle accelerated. Above, the unreinforced sample with an extrusion temperature of 250 °C belonging to experiment number 3 is seen. The rough fracture in the middle region of this sample shows that adhesion occurred at a very good level. This sample also shows the parameters with the highest tensile strength. The samples numbered 1, 4, and 7 show different numbers of reinforcement layers at the lowest temperature level (210 °C). As in sample number 1, it can be understood that the fractures in these samples are also brittle. The increase in strength here originates from the reinforcement layers. The separation of the reinforcement fibers from within the structure is an indication that the bond between the matrix and the reinforcement is not at the desired level. Sample number 9 is the most resistant of the samples with two-layer reinforcement. In this sample as well, the bonding between the fibers and the matrix was not at sufficient level. However, the porous surface can be interpreted as the adhesion within the matrix being good. Samples numbered 4 and 7 have an extrusion temperature of 210 °C, while sample number 9 has an extrusion temperature of 250 °C. When these samples are compared, it is seen that with the increase in extrusion temperature, the molten PLA filament covers the polyester mesh better.

The fracture surfaces of some bending test samples are given in Figure 14. Bending stress is a stress in which tensile stresses occur at the bottom of the sample and compressive stresses occur at the top. This situation leads to different fracture behaviors below and above the neutral axis in bending samples, unlike tensile test samples. Sample number 1 showed a very brittle fracture similar to the tensile samples with the same number. The image of the bottom part of sample number 3 is an indication that it was subjected to bending stress and showed a relatively high energy absorbing ductile fracture. The samples numbered 4 and 6 show one-layer reinforcement samples produced at the lowest and highest extrusion temperatures. It is seen that the fracture surface of sample number 6, in which the bending strength of the sample increased by approximately 53% with the increase in extrusion temperature, exhibits a ductile fracture. In sample number 4, delamination occurred due to insufficient interlayer adhesion due to the low extrusion temperature [1,31,40]. Interlayer separations are visible in this sample. Sample number 9 is a sample with two-layer reinforcement and produced at the highest extrusion temperature. The fracture in this sample occurs in a ductile manner, but delamination is also observed. The gradual decrease in the flexural stress in the test curve of this sample given in Figure 6c also indicates delamination. It is also seen that the bonding of the polyester fibers with the matrix is insufficient in this sample.

## 4. Conclusions

This study experimentally investigated the effect of monofilament polyester mesh interlayers on the mechanical performance of PLA parts produced by the fused filament fabrication (FFF) method. Tensile and three-point bending tests revealed the interactions between different numbers of reinforcement layers (0, 1, 2) and extrusion temperatures (210 °C, 230 °C, 250 °C) on the mechanical properties.

- The tensile test results indicated that extrusion temperature was the most significant parameter affecting both tensile strength and tensile strain. Insufficient interlayer adhesion at the lowest temperature negatively impacted mechanical properties, while significant improvements in strength and strain values were generally observed with increasing temperature. However, a decrease in tensile strain occurred at the highest temperature (250 °C), possibly due to chemical degradation of the PLA material. Increasing the number of polyester mesh reinforcement layers provided approximately a 57% increase in tensile strength specifically at the lowest extrusion temperature (210 °C). However, the effect of increasing the number of reinforcement layers was limited and even slightly negative at higher temperatures where adhesion improved. This is attributed to the insufficient adhesion between PLA and polyester and thermal degradation of polymers at increasing temperatures.
- The flexural test results similarly showed that extrusion temperature had a dominant effect on both flexural strength and flexural strain. A general increase in both values was observed with increasing temperature, with the highest increase (around 53%) achieved in single-layer reinforced samples at 250 °C. Increasing the number of reinforcement layers increased flexural stress at the lowest extrusion temperature, but this effect was not observed at higher temperatures. Insufficient adhesion between the polyester fibers and the PLA matrix was noted across all temperature levels. In addition, it is thought that the degradation of both PLA and polyester materials at high temperatures is also effective in this result.

Overall, this study demonstrates that the use of monofilament polyester mesh interlayers in the FFF production of PLA presents a potential hybrid manufacturing approach for improving mechanical properties, especially with the appropriate selection of extrusion temperature. This method offers an alternative that not only overcomes the limitations of traditional polymer additive manufacturing but also aligns with the trends of hybrid manufacturing and sustainability. Additionally, multi-materials are the advanced version of composites that have varying gradient properties in a specific direction. Based on the necessary functional performance, the constituent material phases for multi-material walls are selected [11]. The hybrid manufacturing method proposed in this study can be used to produce multi-material structures. However, future research focusing on improving the adhesion between PLA and polyester or different fiber/matrix combinations could fully unlock the potential of this hybrid method and enable the production of higher-performance composite materials.

## Figures and Tables

**Table 1:** Constant printing parameters.

Parameters	
Layer height	0.4 mm
Extrusion width	0.72 mm
Nozzle diameter	0.6 mm
Printing speed	40 mm/s
Bed temperature	60 °C
Printing pattern	Rectilinear 90°
Shell outlines	3

**Table 2:** Standards used for mechanical tests and specimen dimensions.

Test Type	ASTM Standard	Length (mm)	Width (mm)	Thickness (mm)	Test Speed (mm/min)
Tensile	D638 [25]	165	13	3.2	5
Three-point bending	D790 [26]	127	12.7	3.2	5

**Table 3:** Experimental design.

Experiment No.	Number of Reinforcement Layers	Extrusion Temperature (°C)
**1**	0	210
**2**	0	230
**3**	0	250
**4**	1	210
**5**	1	230
**6**	1	250
**7**	2	210
**8**	2	230
**9**	2	250

**Table 4:** Calculated mechanical properties of reinforcement monofilament polyester.

Number of Yarns	Ultimate Force (N)	Yarn Tenacity (N)	Tensile Strength (MPa)
**1**	18.52	18.52	589.36
**12**	242.24	20.19	642.56
**13**	263.69	20.28	645.66

**Table 5:** Mechanical properties calculated from tensile tests.

Experiment No	Average Tensile Strength (MPa)	Tensile Strength SD	Average Tensile Strain (%)	Tensile Strain SD	Tensile Modulus (MPa)
**1**	17.81	1.63	0.72	0.09	2767
**2**	45.03	0.28	1.87	0.09	2902
**3**	45.80	0.99	1.83	0.03	2848
**4**	24.80	1.20	0.97	0.08	2781
**5**	42.11	0.23	1.90	0.07	2936
**6**	41.75	1.63	1.76	0.22	2875
**7**	27.95	2.30	1.09	0.12	2876
**8**	32.92	1.86	1.42	0.17	2965
**9**	35.53	0.94	1.58	0.10	2953

**Table 6:** ANOVA table for tensile strength values.

Source	DF	Seq SS	Adj MS	F	P	Contribution %
Number of reinforcement layers	2	33.32	16.66	0.43	0.68	4.34
Extrusion temperature	2	579.82	289.91	7.53	0.04	75.58
Residual error	4	153.98	38.50			20.07
Total	8	767.13				100.00

**Table 7:** ANOVA table for elongation at break values.

Source	DF	Seq SS	Adj MS	F	P	Contribution %
Number of reinforcement layers	2	0.04877	0.02	0.49	0.65	3.20
Extrusion temperature	2	1.2758	0.64	12.69	0.02	83.63
Residual error	4	0.20103	0.05			13.18
Total	8	1.52561				100.00

**Table 8:** Mechanical properties calculated from three-point bending tests.

Experiment No	Average Flexural Strength (MPa)	Flexural Strength SD	Average Flexural Strain (%)	Flexural Strain SD
**1**	57.65	0.05	2.41	0.26
**2**	82.58	2.21	3.52	0.07
**3**	88.24	1.35	4.17	0.14
**4**	64.06	1.85	2.43	0.12
**5**	82.95	1.52	3.06	0.04
**6**	98.34	0.91	3.89	0.04
**7**	67.32	0.37	2.68	0.13
**8**	80.88	0.66	3.41	0.20
**9**	83.02	1.71	3.42	0.26

**Table 9:** ANOVA table for flexural strength values.

Source	DF	Seq SS	Adj MS	F	P	Contribution %
Number of reinforcement layers	2	51.28	25.64	0.75	0.528	3.99
Extrusion temperature	2	1098.9	549.45	16.14	0.012	85.43
Residual error	4	136.19	34.05			10.59
Total	8	1286.37				100.00

**Table 10:** ANOVA table for flexural strain values.

Source	DF	Seq SS	Adj MS	F	P	Contribution %
Number of reinforcement layers	2	0.09902	0.04951	0.57	0.606	3.19
Extrusion temperature	2	2.65706	1.32853	15.3	0.013	85.62
Residual error	4	0.34738	0.08684			11.19
Total	8	3.10346				100.00

## Data Availability

The original contributions presented in this study are included in the article. Further inquiries can be directed to the corresponding author.
